# Tensile energy dissipation and mechanical properties of the knee meniscus: relationship with fiber orientation, tissue layer, and water content

**DOI:** 10.3389/fbioe.2023.1205512

**Published:** 2023-05-31

**Authors:** Andy Morejon, Pedro L. Dalbo, Thomas M. Best, Alicia R. Jackson, Francesco Travascio

**Affiliations:** ^1^ Department of Mechanical and Aerospace Engineering, University of Miami, Coral Gables, FL, United States; ^2^ School of Mechanical Engineering, Georgia Institute of Technology, Atlanta, GA, United States; ^3^ Department of Biomedical Engineering, University of Miami, Coral Gables, FL, United States; ^4^ Department of Orthopedic Surgery, University of Miami, Coral Gables, FL, United States; ^5^ UHealth Sports Medicine Institute, Coral Gables, FL, United States; ^6^ Max Biedermann Institute for Biomechanics at Mount Sinai Medical Center, Miami Beach, FL, United States

**Keywords:** mechanics, quasi-static, dynamic modulus, strength, viscoelasticity, surface layers

## Abstract

**Introduction:** The knee meniscus distributes and dampens mechanical loads. It is composed of water (∼70%) and a porous fibrous matrix (∼30%) with a central core that is reinforced by circumferential collagen fibers enclosed by mesh-like superficial tibial and femoral layers. Daily loading activities produce mechanical tensile loads which are transferred through and dissipated by the meniscus. Therefore, the objective of this study was to measure how tensile mechanical properties and extent of energy dissipation vary by tension direction, meniscal layer, and water content.

**Methods:** The central regions of porcine meniscal pairs (*n* = 8) were cut into tensile samples (4.7 mm length, 2.1 mm width, and 0.356 mm thickness) from core, femoral and tibial components. Core samples were prepared parallel (circumferential) and perpendicular (radial) to the fibers. Tensile testing consisted of frequency sweeps (0.01–1Hz) followed by quasi-static loading to failure. Dynamic testing yielded energy dissipation (*ED*), complex modulus (E*), and phase shift (*δ*) while quasi-static tests yielded Young’s Modulus (*E*), ultimate tensile strength (*UTS*), and strain at *UTS* (*ε_UTS_*). To investigate how *ED* is influenced by the specific mechanical parameters, linear regressions were performed. Correlations between sample water content (*φ_w_*) and mechanical properties were investigated. A total of 64 samples were evaluated.

**Results:** Dynamic tests showed that increasing loading frequency significantly reduced *ED* (*p* < 0.05). Circumferential samples had higher *ED*, E*, *E*, and *UTS* than radial ones (*p* < 0.001). Stiffness was highly correlated with ED (R^2^ > 0.75, *p* < 0.01). No differences were found between superficial and circumferential core layers. *ED*, E*, *E*, and *UTS* trended negatively with *φ_w_* (*p* < 0.05).

**Discussion:** Energy dissipation, stiffness, and strength are highly dependent on loading direction. A significant amount of energy dissipation may be associated with time-dependent reorganization of matrix fibers. This is the first study to analyze the tensile dynamic properties and energy dissipation of the meniscus surface layers. Results provide new insights on the mechanics and function of meniscal tissue.

## Introduction

The human menisci are crescent-shaped fibrocartilaginous tissues within the knee joint that are responsible for load distribution, transmission, and dampening caused by everyday activities including walking and running. In fact, up to 75% of knee loads are borne by the menisci (Shrive et al., 1978; Walker et al., 2015). Accordingly, the primary role of this tissue is to protect the underlying articular cartilage against excessive stress concentration which can result in cartilage wear and, ultimately, the development of knee osteoarthritis (Lohmander et al., 2007).

The particular composition and structure of the meniscus allow the tissue to perform its primary function of dampening mechanical loads at the knee (Gaugler et al., 2015). The tissue is composed of approximately 70% interstitial fluids (Makris et al., 2011) with the remaining volume represented by a porous viscoelastic extracellular matrix (ECM) (Fithian et al., 1990; Morejon et al., 2021b; Norberg et al., 2021; De Rosa et al., 2022; Schwartz et al., 2022). The main component of the ECM is collagen fibers (75% of dry mass), which provide structural integrity (Williamson et al., 2001). Another component of the ECM is a network of negatively charged glycosaminoglycans (GAGs) which draw water into the tissue (Athanasiou and Sanchez-Adams, 2009). The intrinsic viscoelastic nature of the meniscus ECM causes energy dissipation during mechanical loading. However, energy dissipation is also caused by friction generated from fluid flow. When forces are applied to the meniscus, deformation of the ECM occurs creating a local increase of interstitial fluid pressure which forces water through the pores of the matrix. In turn, frictional drag between the fluid and porous matrix is created, which eventually leads to energy dissipation (Mow et al., 2005). Previous works have shown that the extent of energy dissipated by the meniscus is related to factors such as loading frequency, tissue water content, and meniscal region (Gaugler et al., 2015; Morejon et al., 2022).

Although knee loads often arise in the form of axial compression, they are transferred into hoop stresses via Poisson effects (Fox et al., 2012), resulting in significant tensile strains (Kolaczek et al., 2016). The meniscus core is highly organized with collagen fibers primarily aligned along the length of the tissue known as circumferential fibers. However, a much lower density of fibers is also aligned in the radial orientation (Skaggs et al., 1994; Petersen and Tillmann, 1998). Evidence of greater stiffness and strength along the circumferential orientation of the tissue shows that circumferential fibers enhance the mechanical properties to resist such hoop stresses (Proctor et al., 1989; Leroux and Setton, 2002; Li et al., 2005; Lakes et al., 2015; Peloquin et al., 2016). However, inhomogeneity exists in the macrostructure of the meniscus between the core and superficial layers (Choi et al., 2016; Andrews et al., 2017). Unlike the highly aligned core region, surface layers contain a mesh-like structure with collagen fibers randomly oriented in a 2-D network (Petersen and Tillmann, 1998; Sweigart and Athanasiou, 2005; Bryceland et al., 2017). Our previous work investigating meniscal shear properties demonstrated significant differences in complex modulus between the core and femoral (superior) and tibial (inferior) superficial layers (Schwartz et al., 2022). Whether similar findings occur between tissue layers in tensile loading is unknown.

Accordingly, we investigated the role of fiber orientation and tissue layer (i.e., femoral, tibial, and core) on tensile energy dissipation and mechanical properties of the meniscus. In addition, we hypothesize that the tissue’s capacity to dissipate energy is affected by its material characteristics including viscoelasticity and stiffness. Hence, the relationship between energy dissipation and tensile mechanical parameters was analyzed. Furthermore, the relationship between mechanical parameters and energy dissipation was examined. Amount of water contained in the tissue has been shown to correlate negatively with mechanical properties in compression and shear (Bursac et al., 2009a; Norberg et al., 2021; Schwartz et al., 2022). Accordingly, this study explored possible relationships between water content and tensile mechanical properties. It was hypothesized that the tensile behavior is significantly affected by the tissue’s orientation, layer structure, and water content. To test this hypothesis, tensile meniscal samples from different layers of the tissue were subjected to dynamic and quasi-static loading parallel and perpendicular to fiber orientation. Finally, samples were dried and weighed to relate their water content to mechanical properties.

## Materials and methods

### Sample preparation

**FIGURE 1:**
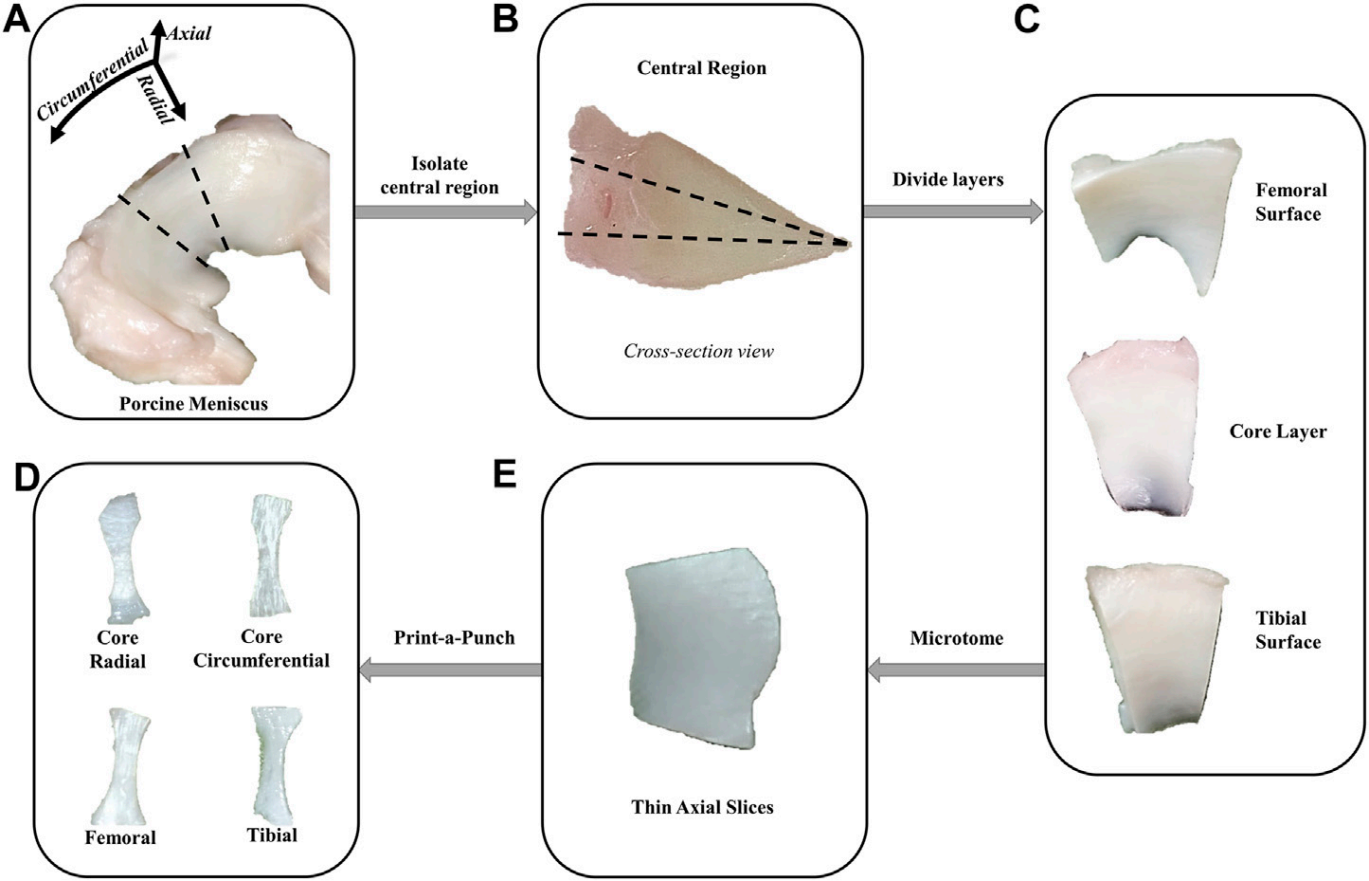
Sample preparation methodology. **(A)** The central regions of porcine menisci were isolated. **(B, C)** A razorblade was used to isolate the superficial surfaces from the core layer. **(D)** Next, a freezing stage microtome was used to obtain thin axial slices from each layer. The femoral and tibial samples were only obtained from the surface of their corresponding layers. **(E)** Print-a-punch tool was used to cut slices into dog bone samples in radial or circumferential orientations.

Eight porcine meniscal pairs (n = 8) were obtained from a commercial tissue source (2+ years old, Animal Technologies, Tyler, TX). Tissue central regions were isolated for testing and cut parallel to the superficial layers to ensure that the femoral and tibial surfaces could be placed flat on a cutting platform. A freezing stage microtome (SM2010 R, Leica Biosystems, Deer Park, IL) was used to cut the tissue into thin slices in the axial plane. Two slices were harvested from the core and one slice from the superior (femoral) and inferior (tibial) surfaces. A custom 3D printed Print-A-Punch tool, similar to that described in the literature (Nelson et al., 2020), was used to cut slices into dumbbell-shaped samples along two orientations. Since the main contribution of the fibers is in the circumferential orientation (Zhu et al., 1994), core slices were cut parallel (circumferential direction of meniscus) or perpendicular (radial direction of meniscus) to the fibers. Femoral and tibial samples were only cut along the circumferential direction given that there is no preferential fiber orientation in those layers, see Figure 1 (Proctor et al., 1989). A total of 32 samples were prepared: 16 from the core (8 circumferential and 8 radial), 8 from the femoral surface, and 8 from the tibial surface. Samples were stored frozen at −20°C in protease-inhibited phosphate buffered saline (PBS) until testing to avoid degradation. Immediately prior to mechanical testing, specimens were thawed for 30 min and measured with a micrometer (Mitutoyo, Kanagawa, Japan) to record their width (2.1 ± 0.1 mm) and thickness (0.35 ± 0.13 mm).

### Dynamic testing

Dynamic tension tests were performed to characterize the tissue’s dynamic mechanical properties and energy dissipation. To maintain sample physiological hydration during testing, PBS was periodically sprayed onto specimens (Lakes et al., 2015). Tissue samples were loaded onto an uniaxial mechanical tester equipped with a 200 N load cell (ElectroForce 5,500 series, TA Instruments, New Castle, DE). A 0.1 N preload was applied before the sample length (4.7 ± 0.8 mm) was measured using digital calipers (Model 500-196-30, Mitutoyo). This initial load was applied to ensure that the test sample was taut prior to dynamic loading (Maritz et al., 2021). A 10% strain tensile frequency sweep was performed increasing from 0.01 to 1 Hz for 10 cycles each (i.e., 0.01, 0.05, 0.1, 0.25, 0.5 and 1 Hz). This range of loading frequencies was selected to simulate physiological loading conditions such as walking (Tissakht and Ahmed, 1995; Choi et al., 2016).

Dynamic testing assessed for energy dissipation (*ED*), tensile complex modulus (E*), and phase shift (*δ*) across all frequencies. A custom MATLAB^®^ script (v2022a, MathWorks Inc., Natick, MA) was used to calculate these dynamic mechanical parameters. Specifically, *ED* was quantified as the integral of the hysteresis cycle, and E* as peak stress divided by peak strain (Abdel-Sayed et al., 2014; Morejon et al., 2022). Meanwhile *δ* was computed as the time interval between peak stress and peak strain, divided by cycle length (Vriend and Kren, 2004).

### Quasi-static testing

**FIGURE 2:**
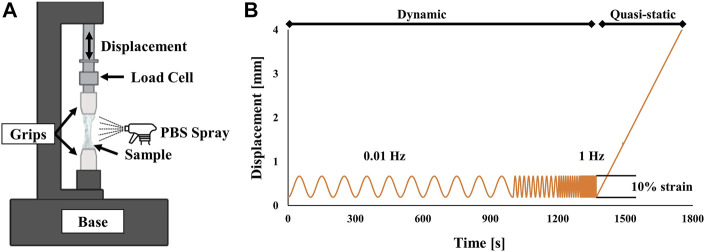
**(A)** Schematic of experimental test setup. Samples were held by aluminum grips and sprayed with PBS during the experiment to remain hydrated. **(B)** Experimental loading conditions. Dynamic frequency sweeps were performed from 0.01 to 1 Hz followed by a quasi-static pull until failure.

Quasi-static mechanical properties were recorded using load to failure tension testing. Once the frequency sweep was complete, samples were stretched to failure at a rate of 0.01 mm/s in quasi-static tension. A low strain rate was selected to ensure that no viscoelastic effects were present, see Figure 2 (Warnecke et al., 2020; Maritz et al., 2021).

A MATLAB^®^ script was used to calculate Young’s Modulus (*E*), ultimate tensile stress (*UTS*), and strain at *UTS* (*ε*_*UTS*_). Specifically, *E* was computed from the linear region of the stress-strain curve. *UTS* was calculated from the peak stress, while *ε*_*UTS*_ was the corresponding strain at peak stress.

### Water content measurements

Water content of meniscal samples was measured as the mass fraction with respect to the weight of the hydrated sample. Mass was recorded before and after lyophilization using an analytical scale (Model ML, Mettler Toledo, Columbus, OH). Percent water content (*φ*_*w*_) was calculated based on the following equation:

$$\varphi_{w}=\frac{W_{wet}-W_{dry}}{W_{wet}}\;X\;100\%$$

where *W*_*wet*_ and *W*_*dry*_ are the wet and dry weights of the samples, respectively.

### Statistical analysis

Statistical tests were performed using Minitab Statistical Software (Version 20.2, State College, PA). Grubb’s tests were used to eliminate outliers. Data normality was confirmed via Anderson-Darling test. A three-way ANOVA investigated the main effects and interactions of frequency, meniscal layer/orientation, and compartment (medial vs. lateral) on ED, E*, and *δ*. Since quasi-static tension is frequency independent, a two-way ANOVA was used to analyze the main effects and interactions of meniscal layers/orientations and compartments on *E*, *UTS*, and *ε*_*UTS*_. Furthermore, Tukey post-hoc tests were conducted to find statistically significance differences between individual group comparisons. In addition, simple linear regression analyses quantified the relationship between *ED*, as dependent variable, and each of the mechanical parameters (E*, *δ*, *E*, *UTS*, and *ε*_*UTS*_) individually, as regressors. To explore these relationships, all the frequency values tested (*ED*, E*, and *δ*) were averaged to obtain a single dynamic parameter value.

In addition, a two-way ANOVA investigated for potential significant interactions and main effects (compartment and layer) on *φ*_*w*_. Furthermore, possible relationships between the tissue’s water content and its mechanical properties were investigated. Linear regression analyses were used to analyze the relationship between *φ*_*w*_ and mechanical parameters (i.e., *ED*, E*, *δ*, *E*, *UTS*, and *ε*_*UTS*_).

For all statistical tests performed, the level of significance was set to *α* = 0.05. All data are reported as mean ± standard deviation.

## Results

### Dynamic properties

**FIGURE 3:**
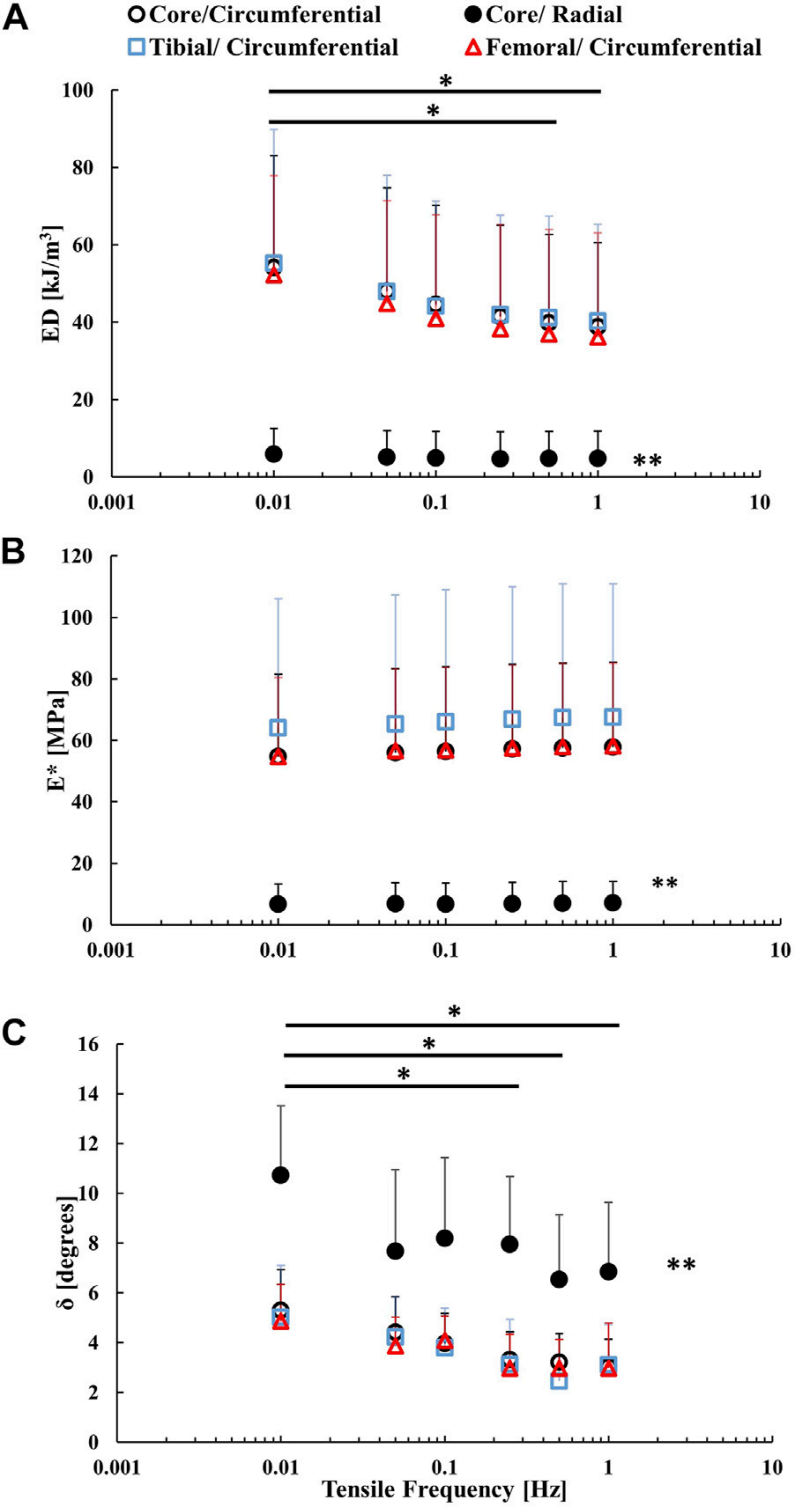
Frequency sweeps of **(A)** complex modulus **(** E*), **(B)** energy dissipation (*ED*), and **(C)** phase shift (*δ*) by sample orientation and layer. Medial and lateral samples were pooled together. Statistical significance between frequency levels is indicated by * (*p* < 0.05). Statistical significance between the radial samples and the remaining groups is denoted by ** (*p* < 0.001).

Figure 3 *ED* was significantly reduced by increasing loading frequency (*p* < 0.05). Specifically, *ED* at 0.01 Hz was significantly higher than at 0.5 and 1 Hz (*p* < 0.05). Load dampening by the tissue was also greater in circumferential samples as evidenced by their significantly greater *ED* compared to radial samples (*p* < 0.001). There was no effect of layer (femoral, tibial, and core) or compartment (medial and lateral) on *ED*.

There was a slight increasing trend for the complex modulus, E*, with load frequency, but this was not statistically significant (*p* = 0.642). E* was significantly higher in circumferential samples versus radial ones (*p* < 0.001). Comparisons between compartment (medial and lateral) and layer (circumferential core, femoral, and tibial) revealed no significant main effects or interactions on E*. There was a decreasing trend for phase shift *δ* for increasing load frequency, with significant differences discovered between the 0.01 Hz and the 0.25, 0.5, and 1 Hz frequency groups. Radial samples had a significantly greater *δ* (8.2° ± 2.4°) than circumferential samples (3.7° ± 1.1°, *p* < 0.001). However, no significant effects of layer and compartment were found on *δ*.

### Quasi-static properties

Table 1 In the core layer, *E* of circumferential samples averaged 122.6 ± 49.6 MPa and was significantly larger than the average for radial samples (9.8 ± 7.8 MPa; *p* < 0.001). On the other hand, *E* did not significantly vary between tibial (132.8 ± 80.3 MPa), femoral (135.0 ± 59.9 MPa) and circumferential core (122.6 ± 49.6 MPa) samples. Similarly, no differences were found between medial and lateral compartment stiffnesses (*p* = 0.653).

**TABLE 1:** Quasi-static tension results separated by sample layer and orientation.

Layer	Orientation	Compartment	E [MPa]	UTS [MPa]	ε_UTS_ [%]
Core	Radial	Medial	13.9 ± 9.1	2.2 ± 1.1	25.0 ± 10.7
		Lateral	6.2 ± 4.2	1.3 ± 0.8	40.5 ± 12.9
		Average	9.8 ± 7.8	1.7 ± 1.0	31.7 ± 13.8
	Circumferential	Medial	132.8 ± 38.1	27.5 ± 11.3	28.7 ± 6.8
		Lateral	112.4 ± 59.8	20.3 ± 9.9	33.8 ± 9.5
		Average	122.6 ± 49.6	23.9 ± 10.9	31.3 ± 8.4
Femoral		Medial	118.6 ± 72	25.1 ± 15.3	33.5 ± 7.7
		Lateral	151.5 ± 43.5	31.3 ± 12.6	33.2 ± 4.6
		Average	135.0 ± 59.9	28.2 ± 13.9	33.4 ± 6.1
Tibial		Medial	116.2 ± 80.7	23.8 ± 15.3	31.1 ± 5.6
		Lateral	149.4 ± 81.7	28.7 ± 12.7	31.9 ± 8.2
		Average	132.8 ± 80.3	26.3 ± 13.8	31.5 ± 6.8

The *UTS* of meniscal tissue was significantly higher (*p* < 0.01) in circumferential samples (23.9 ± 10.9 MPa) versus radial ones (1.7 ± 1.0 MPa). However, no significant variations in *UTS* were discovered between tibial (26.3 ± 13.8 MPa), femoral (28.2 ± 13.9 MPa) and circumferential core samples. Also, compartments (i.e., medial and lateral) were not statistically different in *UTS*. For *ε*_*UTS*_, neither sample orientation nor tissue layer had a statistically significant effect. However, the compartment and its interaction with layer did affect *ε*_*UTS*_. In the core layer, lateral meniscal samples (40.5% ± 12.9%) reached significantly larger *ε*_*UTS*_ than their medial (25.0% ± 10.7%) counterparts (*p* < 0.05).

### ED vs. mechanical parameters

Table 2 The relationships between average energy dissipation and tensile mechanical parameters (i.e., E*, *δ*, *E*, *UTS*, and *ε*_*UTS*_) were investigated. Since no effect of layer was observed in the mechanical parameters, circumferential samples from all layers (i.e., femoral, tibial, and core) were pooled. Average *ED* had a strong positive correlation in both orientations for E* (*p* < 0.001, *R* ^*2*^ = 0.863) and E (*p* < 0.001, *R* ^*2*^ = 0.750). Moreover, negative correlations were discovered between *δ* and *ED*. This association was stronger in radial samples (*p* = 0.001, *R* ^*2*^ = 0.596) than in circumferential samples (*p* < 0.001, *R* ^*2*^ = 0.301). In *UTS*, the trend with *ED* was also positive but stronger in circumferential samples (*p* < 0.001, *R* ^*2*^ = 0.646) versus radial ones (*p* = 0.012, *R* ^*2*^ = 0.349). Finally, no significant correlation was discovered between *ED* and *ε*_*UTS*_ (*R* ^*2*^ = 0.099).

**TABLE 2:** Linear regression results between energy dissipation (*ED*) and tensile mechanical properties (E*, *δ*, *E*, *UTS*, and *ε*_*UTS*_) divided by sample orientation. Samples cut in circumferential direction from femoral, tibial and core layers were pooled together. Statistically significant correlations denoted by *.

Parameter	Sample orientation	*p*-value	*R* ^*2*^	Relationship
E*	Circumferential	<0.001 *	0.863	+
	Radial	<0.001 *	0.984	+
*δ*	Circumferential	<0.001 *	0.301	-
	Radial	0.001 *	0.596	-
*E*	Circumferential	<0.001 *	0.750	+
	Radial	<0.001 *	0.835	+
*UTS*	Circumferential	<0.001 *	0.646	+
	Radial	0.012 *	0.349	+
*ε*_*UTS*_	Circumferential	0.911	0.000	-
	Radial	0.145	0.099	-

### Water content

**FIGURE 4:**
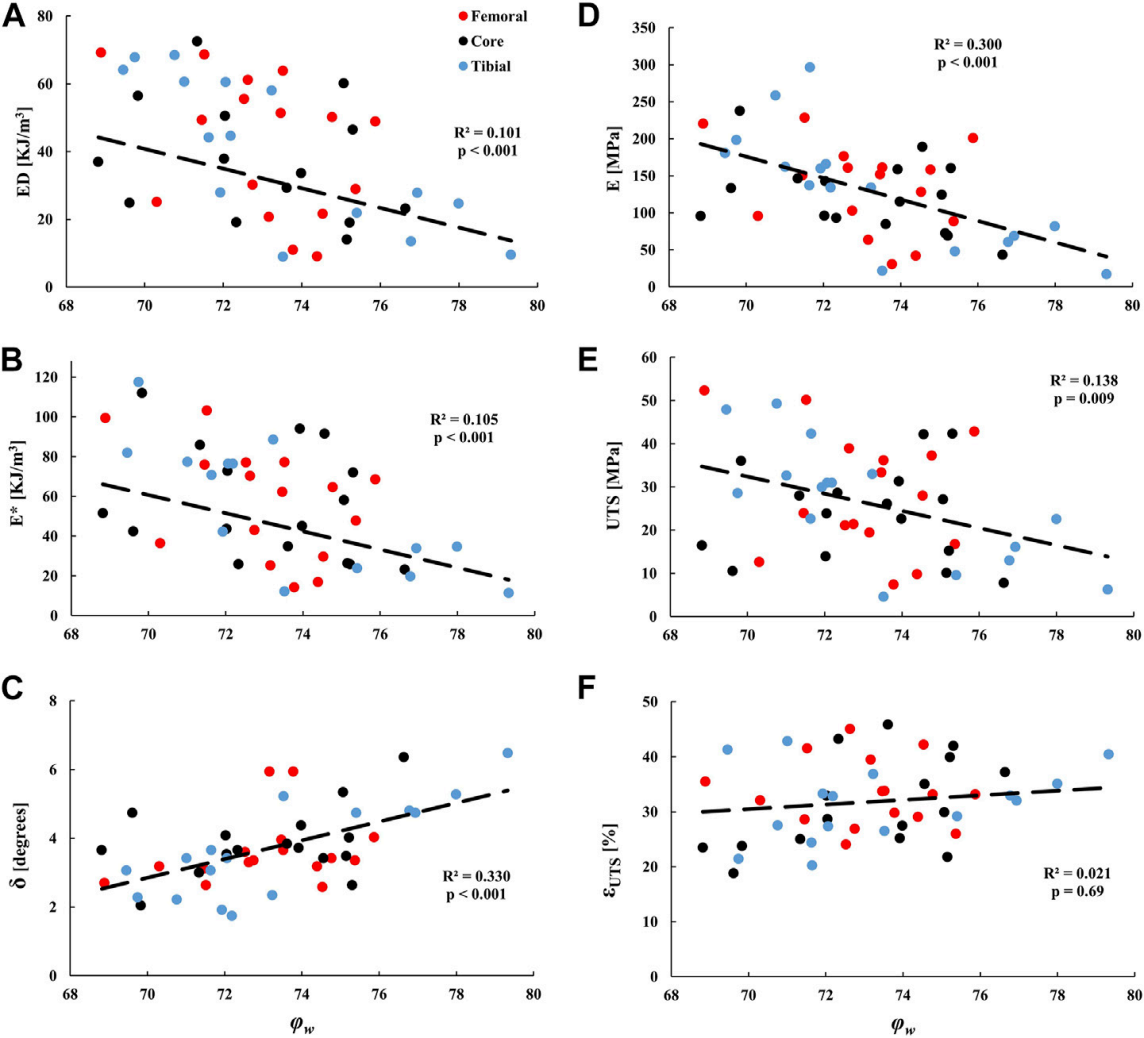
Linear regression analyses of the tissue water content (*φ*_*w*_) against the tensile dynamic parameters: **(A)** *ED*, **(B)** E*, and **(C)** *δ*; and quasi-static parameters: **(D)** *E*, **(E)** *UTS*, and **(F)** *ε*_*UTS*_ of samples from the tibial, femoral, and core circumferential layers. Medial and lateral compartments were pooled together.

Table 3 Average *φ*_*w*_ was 73.5% ± 2.3% across 64 samples. No significant differences in *φ*_*w*_ were found across meniscal compartments and layers. The effect of tissue hydration on mechanical properties was also analyzed. Radial samples originating from the core were treated separately in the analysis due to the large effect fibers had on the mechanical parameters. Mechanical properties (i.e., *ED*, E*, *δ*, *E*, *UTS*, and *ε*_*UTS*_) of radial samples did not correlate with *φ*_*w*_. On the other hand, *φ*_*w*_ of circumferentially cut samples from all layers had weak negative correlations with *ED*, E*, *E*, and *UTS*, (*p* < 0.01, 0.10 < *R* ^*2*^ < 0.30) and weak positive correlations with *δ* (*p* < 0.001, *R* ^*2*^ = 0.330), see Figure 4.

**TABLE 3:** Meniscal water content by compartments and layers.

Meniscal compartment/Layer	Water content, *φ*_*w*_ [%]
Total (*n* = 64)	73.5 ± 2.3
***Medial (*n *= 32)***	*73.8 ± 2.0*
Core (*n* = 16)	74.1 ± 1.8
Femoral (*n* = 8)	73.5 ± 1.3
Tibial (*n* = 8)	73.7 ± 2.9
***Lateral (n =32***)	***73.2 ± 2.6***
Core (*n* = 16)	73.7 ± 2.5
Femoral (*n* = 8)	72.6 ± 2.3
Tibial (*n* = 8)	73.0 ± 3.3

## Discussion

Previous studies have characterized the tensile quasi-static response of the meniscus (Proctor et al., 1989; Leroux and Setton, 2002; Abdelgaied et al., 2015; Fischenich et al., 2015; Lakes et al., 2015; Warnecke et al., 2020). Herein, we extended this work by investigating both the quasi-static and dynamic tensile mechanical behavior of meniscal tissue across loading orientations, tissue layers, and compartments. Shear, compressive, and tensile mechanical properties have been previously investigated across different meniscal layers (Proctor et al., 1989; Tissakht and Ahmed, 1995; Moyer et al., 2012; Freutel et al., 2015; Choi et al., 2016; Maritz et al., 2021; Schwartz et al., 2022). However, to our knowledge, this is the first study to analyze the tensile dynamic properties and quantify energy dissipation in the superficial and core layers of the meniscus thereby moving closer to a clinical approximation of everyday joint and tissue loading.

A dynamic test battery showed that *ED* was inversely related to loading frequency in circumferentially oriented samples (*p* < 0.05). Previous studies reported a similar effect in cyclic compression (Morejon et al., 2021a; Morejon et al., 2022). Hence, the capacity of the meniscus to dissipate mechanical energy is time-dependent and reduced during high-frequency loading such as in the case of impact loading (Gaugler et al., 2015). This finding may result from the intrinsic viscoelasticity of the collagen fibers which rearrange in a time dependent manner (Han et al., 2018).

At all frequencies, *ED* was significantly larger along the direction parallel to the fibers (*p* < 0.05). After normalizing for load strain, energy dissipation was found to be in the range of previous studies under tensile loading in both orientations (Freutel et al., 2015; Lakes et al., 2015; Lakes et al., 2016). Dynamic tests measured *ED* of circumferential samples to average 44.5 kJ/m^3^ while radial samples averaged 5.0 kJ/m^3^ of energy dissipation. This implies that collagen fibers further enhance the tissue’s ability to dissipate mechanical loads. Nonetheless, in samples loaded perpendicular to the fibers, energy dissipation was ten-fold higher in tension than in compression (0.5 kJ/m^3^) at similar strains and frequencies (Morejon et al., 2021a; Morejon et al., 2022). The mechanism of energy dissipation in tension is believed to occur via time-dependent reorganization of the matrix fibers, while in compression, it occurs primarily by fluid flow friction (Park and Ateshian, 2006; Morejon et al., 2022). Hence, the results of this study show that the most efficient energy dissipation mechanism in the meniscus is associated with time-dependent reorganization of the matrix fibers (Gaugler et al., 2015).

Complex modulus, E* values increased slightly with load frequency but the effect was not statistically significant. A previous study by Bursac et al. on human menisci observed similar findings in tensile dynamic modulus ranging from 0.01 to 1 Hz (Bursac et al., 2009b). In the present study, average E* was significantly higher in circumferential (∼57 MPa) versus radial orientation (∼7 MPa). However, measurements of circumferential dynamic modulus by Bursac et al. ranged from 115 - 143 MPa, which are higher but within the same order of magnitude as the present study (Bursac et al., 2009b). This difference could be attributed to the species and region of the samples used. Bursac et al. used human samples from the outer third region of the meniscus as opposed to the present study which employed the central region of porcine menisci (Bursac et al., 2009b).

Phase shift, *δ*, significantly decreased with loading frequency (*p* < 0.05). This trend is consistent with previous studies in dynamic compression (Bursac et al., 2009a; Pereira et al., 2014; Coluccino et al., 2017), and shear (Norberg et al., 2021; De Rosa et al., 2022; Schwartz et al., 2022) at similar frequencies. The phase shift is commonly associated with mechanical energy dampening (Park and Ateshian, 2006; Bursac et al., 2009a; Pereira et al., 2014; Danso et al., 2015; Gaugler et al., 2015; Han et al., 2018). This study confirms that *δ* follows a similar frequency-dependent pattern to *ED*. Direction of loading had a significant effect on *δ* (radial: 8.2°, circumferential: 3.7°, *p* < 0.001), suggesting that the presence of collagen fibers reduced the viscous effect compared to the raw ground matrix.

Average *E* of meniscal samples in quasi-static loading was greater along the fiber direction (*p* < 0.001) and of similar magnitude to previous measurements on porcine menisci in circumferential and radial orientations (Abdelgaied et al., 2015; Lakes et al., 2015; Lakes et al., 2016; Maritz et al., 2021). Our findings confirm that circumferential fibers greatly enhance tensile stiffness to resist high hoop stresses produced by daily mechanical loading (Daszkiewicz and Łuczkiewicz, 2021). Also, mean *UTS* for circumferential samples, 23.9 MPa, was greater than the 1.7 MPa measured in radial specimens (*p* < 0.001). These values are similar to those from previous testing performed for both orientations on porcine menisci (Abdelgaied et al., 2015; Lakes et al., 2016). Therefore, collagen fibers may aid in meniscal tear prevention by reinforcing tissue strength in the circumferential orientation. *ε*_*UTS*_ averaged ∼32% which is comparable to values found in previous studies employing tensile loading (Freutel et al., 2015; Lakes et al., 2015; Lakes et al., 2016; Peloquin et al., 2016; Creechley et al., 2017; Peloquin et al., 2018). Differences in *ε*_*UTS*_ were discovered between medial and lateral meniscal compartments (*p* < 0.05). Similarly, in a previous study in human samples, maximum strain was also larger on average in the lateral compartment but differences were not statistically significant (Tissakht and Ahmed, 1995).

*ED* was positively correlated with E*, *E*, and *UTS* (*R* ^*2*^ > 0.35). This result implies that the capability of energy dissipation by the tissue depends largely on its ECM’s mechanical stiffness and strength. Collagen fibers are not only associated with providing high tensile strength, since they are also intrinsically viscoelastic (Gautieri et al., 2012; Sopakayang et al., 2012). Therefore, besides strength and stiffness, collagen fibers may also increase the capacity of the meniscus to dissipate energy. Average *δ* had a negative correlation with *ED* (*R* ^*2*^ = 0.3–0.6) suggesting that the association of *δ* with *ED* may not be direct when comparing across tissue samples. However, it remains useful for estimating the effect of frequency on energy dissipation when measuring phase shift (Park and Ateshian, 2006; Bursac et al., 2009a; Pereira et al., 2014; Danso et al., 2015; Gaugler et al., 2015).

No significant differences existed between the three tissue layers in *ED*, E*, *δ*, *E*, *UTS,* or *ε*_*UTS*_. Previous studies similarly observed no significant differences in circumferential tensile mechanical parameters between superficial and core tissue layers (Tissakht and Ahmed, 1995; Freutel et al., 2015). Suggesting that the mesh-like organization of the surface layer fibers yields a similar tensile response to the core along its fiber-aligned orientation. However, other studies have reported conflicting evidence about the tensile properties of the core in the circumferential orientation: one study found it to be more compliant (Maritz et al., 2021), while another found it to be stiffer (Proctor et al., 1989) than superficial layers. The tensile properties reported in the literature may be affected by sample dimension, species, and specific region of collection (Lechner et al., 2000). Therefore the discrepancies seen between the present study and others may be a result of variations between the aforementioned factors (Proctor et al., 1989; Maritz et al., 2021). Further investigation is needed to verify if the structural differences between the three layers lead to differences in tensile mechanical properties.

Samples evaluated in the current study had an average *φ*_*w*_ of 73.5% and this was not statistically different across meniscal compartment and layer. Degree of water content for our samples is comparable with previous measurements of porcine menisci (Schwartz et al., 2022). Also, *φ*_*w*_ had a weak negative correlation with *ED* (*R* ^*2*^ = 0.101). A similar relationship was seen in compressive energy dissipation measurements under similar loading strains and frequencies (Morejon et al., 2021a; Morejon et al., 2022). Weak negative correlations were also obtained between *φ*_*w*_ and E*, *E*, and *UTS* of circumferential samples from all layers (*p* < 0.01). A similar trend was noted in previous studies wherein water content correlated with reduced stiffness parameters and energy dissipation in compression and shear (Joshi et al., 1995; Bursac et al., 2009a; Seitz et al., 2013; Norberg et al., 2021; De Rosa et al., 2022; Schwartz et al., 2022). A possible explanation for this trend could be that samples with a higher water content contain less ECM mass which is known to provide the tissue with its mechanical integrity (Athanasiou and Sanchez-Adams, 2009). Therefore, samples with lower ECM to water ratios tend to be mechanically weaker. Finally, *δ* had a positive correlation with *φ*_*w*_ in circumferential samples (*R* ^*2*^ = 0.330). This indicates that the tissue’s intrinsic viscoelastic properties tend to increase with tissue hydration.

There are some limitations in the present study. First, the tissues were derived from porcine as opposed to human menisci. However, porcine tissue has been widely used as an animal model approximating human tissue whereby similar magnitudes and trends in mechanical behavior have been found between the two species (Joshi et al., 1995; Sweigart et al., 2004; Norberg et al., 2021). Secondly, testing frequency did not exceed 1 Hz due to limitations in accurate data collection at higher frequencies. Importantly, higher loading rates may be experienced by the meniscus during running or jumping. Also, only central regions of the menisci were investigated in the present study. The differences in mechanical properties between central and horn regions have been extensively investigated with some major differences noted (Kleinhans and Jackson, 2018; Seitz et al., 2021; Morejon et al., 2022). Finally, samples employed were cut in the radial and circumferential orientations of the meniscus but not in the vertical or axial direction. Variations in the material parameters have been previously observed in compression studies (Berni et al., 2021). However, since both radial and axial directions are perpendicular to the circumferential fibers, we have limited our samples to only one of these. Hence, future studies should focus on addressing the aforementioned limitations by sampling all regions and orientations of the tissue and/or testing at higher loading frequencies.

## Conclusion

The main finding of this study was that tensile energy dissipation and mechanical properties of meniscal tissue are highly dependent on loading direction. Importantly, circumferential fibers stiffen, strengthen, and increase the energy dissipating capability of the meniscus. Conversely, surface layers were not mechanically different from tissue core evaluated in the circumferential orientation. Moreover, energy dissipation was positively correlated to its stiffness and negatively related to water content. Finally, water content was weakly associated with tensile mechanical properties of samples loaded parallel to fibers. Overall, this study improves our understanding of the tensile mechanical behavior of meniscal tissue. To effectively replicate the mechanical behavior of native meniscus, replacement surrogates should focus primarily on mimicking the fiber-orientation. The large discrepancy in the tensile properties between the radial and circumferential orientations play a critical role in the functioning of the tissue.

## Data Availability

The raw data supporting the conclusion of this article will be made available by the authors, without undue reservation.
